# Insights into the Composition and Function of Virus Communities During Acetic Acid Fermentation of Shanxi Aged Vinegar

**DOI:** 10.3390/foods14173095

**Published:** 2025-09-03

**Authors:** Zhen Yu, Huizi Zhao, Tingting Ma, Xujiao Zhang, Yufeng Yan, Yini Zhu, Yongjian Yu

**Affiliations:** 1 Shanxi Provincial Key Laboratory for Vinegar Fermentation Science and Engineering, Shanxi Zilin Vinegar Industry Co., Ltd., Taiyuan 030400, China; yuzhen@just.edu.cn (Z.Y.); 18435993458@163.com (H.Z.); 15235168247@163.com (X.Z.); 2 School of Grain Science and Technology, Jiangsu University of Science and Technology, Zhenjiang 212003, China; 18861754137@163.com (T.M.); susu052199@163.com (Y.Z.)

**Keywords:** vinegar, virus, viral metagenomics, auxiliary metabolic genes, fermented foods

## Abstract

Viruses play a regulatory role in microbial ecology. Traditional fermented foods have complex fermentation environments with abundant viral participation, yet current research on viral communities in fermented foods remains insufficient. Traditional, manually produced solid-state fermented vinegar serves as an excellent model for studying the role of viral communities in fermented foods. Using metagenomic approaches, this study investigates the structure and dynamics of viral communities during the acetic acid fermentation process of Shanxi aged vinegar. All identified viruses were bacteriophages, and the dominant families were identified as Herelleviridae, Autographiviridae, and Stanwilliamsviridae. The richness and diversity of viral communities exhibited significant variations during acetic acid fermentation. Furthermore, correlation analysis revealed a strong association (*p* < 0.01) between core bacteria and core viruses. Functional annotation revealed the presence of viral genes associated with amino acid and carbohydrate metabolism. Notably, abundant auxiliary carbohydrate-active enzyme (CAZyme) genes were identified in viruses, with glycoside hydrolases (GHs), glycosyltransferases (GTs), and carbohydrate-binding modules (CBMs) demonstrating particularly high abundance. Additionally, several antibiotic resistance genes were detected in viruses. This study elucidates the impact of viral communities on microbial dynamics during food fermentation, advancing our understanding of viral roles in traditional fermented food ecosystems.

## 1. Introduction

Viruses represent essential components of natural microbial communities and are ubiquitous across nearly all ecosystems. Viruses can shape host microbial community composition through lysis while also modulating host metabolism by regulating the lytic–lysogenic switch [1]. Based on rough estimates, approximately 20–40% of bacterial cells in aquatic environments are believed to be in a virus-infected state and undergoing virus-directed metabolic reprogramming [2]. The viral genome harbors numerous auxiliary metabolic genes (AMGs), enabling viruses to reprogram host metabolism through these encoded functions. Research demonstrates that viruses can significantly influence various host metabolic processes, including photosynthesis, nucleotide biosynthesis, and the uptake of essential nutrients such as nitrogen, phosphorus, and sulfur [2,3,4]. Viruses can promote host growth through AMGs, as exemplified by prophage-encoded chitinases that enable *Pseudomonas* sp. to degrade chitin and proliferate in chitin-rich environments [5]. Moreover, viruses harbor AMGs associated with biogeochemical cycling, which can enhance these critical earth processes [6,7]. For instance, phage (bacterial viruses)-encoded AMGs have been shown to improve soil carbon sequestration in vermicompost-amended soils [8]. Notably, viruses are increasingly recognized as reservoirs of antibiotic resistance genes (ARGs), capable of conferring enhanced antibiotic resistance to their host organisms [9]. Viruses can enhance host adaptation to environmental abiotic stresses through AMGs [10,11], thereby driving host evolution. The coevolution of viruses and bacteria is recognized as a key driver of microbial community ecology and evolution. While research on viral ecology has expanded significantly, studies of permafrost soil viruses have revealed a substantial number of taxonomically distinct novel viruses that diverge from known environmental viral groups [12,13], indicating most viruses are still unexplored ‘dark matter’ in the natural microbial community.

Given the lytic effects of viruses on their hosts, research on viruses in food industrial production has primarily focused on phage elimination and phage cocktail therapy against pathogenic bacteria [14,15,16]. In contrast, studies on viral communities in traditional fermented foods remain limited, likely due to the inherent complexity of these fermentation ecosystems, which sustain dynamic multispecies consortia of bacteria, fungi, and viruses. Studies on the viromes of Baijiu and its fermentation starter (*Daqu*) have revealed associations between viral communities and liquor flavor profiles [17,18]. Meanwhile, metagenomic analysis of soy sauce fermentation has identified abundant AMGs related to carbohydrate and amino acid metabolism in viral genomes [19]. The viral communities in fermented vegetables were predominantly composed of dsDNA viruses, with their hosts primarily belonging to the *Lactobacillaceae* and *Enterobacteriaceae* families. Notably, the viral community structure varied significantly across different types of fermented vegetables [20]. In cacao bean fermentation, 62 out of 68 identified viruses were novel taxa, further evidencing the widespread existence of ‘viral dark matter’ in traditional fermented foods [21]. Vinegar is the world’s most widely used acidic condiment, with representative varieties including Italian balsamic vinegar, British malt vinegar, and Chinese cereal vinegar (Zhenjiang aromatic vinegar, Shanxi aged vinegar, and Sichuan bran vinegar, among others). However, research on the vinegar fermentation virome remains scarce. Apart from our previous study on Zhenjiang aromatic vinegar [22], only one investigation on liquid-fermented rice vinegar has been reported to date [23]. Shanxi aged vinegar, one of China’s four famous vinegars, is produced in northern China’s Shanxi province using sorghum as the primary raw material [24]. This contrasts sharply with Zhenjiang aromatic vinegar from southern China, which employs glutinous rice as its main substrate [25,26]. Moreover, these two vinegars differ significantly in their fermentation processes. To date, no studies have investigated the viral communities during Shanxi aged vinegar fermentation. Investigating the viral communities during Shanxi aged vinegar fermentation could provide critical insights into the underlying microbiological mechanisms of this traditional fermentation process. Furthermore, geographical traceability is essential for ensuring the quality and safety of fermented foods. Notably, region-specific viral communities in fermented products demonstrate superior accuracy over bacterial communities for determining geographical origins [27]. As a Chinese protected geographical indication product, characterizing the virome of Shanxi aged vinegar may significantly enhance its origin authentication capabilities [28]. In addition, the primary limiting factors for the mechanization of traditional solid-state fermentation vinegar production are the incomplete understanding of the microbial mechanisms behind vinegar fermentation, particularly the role of the virome. In our preliminary studies, it was found that there is a significant difference in quality between vinegar produced using direct-vat sets composed of only a few fermentation bacteria and vinegar made through traditional brewing methods [29]. In the future, based on a clear understanding of the role of viral communities in solid-state fermented vinegar, viruses containing abundant functional genes related to fermentation can be isolated and added to bacterial agents to create composite direct inoculation starters. This strategy aligns with previous research and applications of phages in fermented vegetables [30]. These can then be applied to solid-state vinegar production to accelerate the mechanization and intelligentization of traditional solid-state fermented vinegar brewing.

In this study, we characterize the viral communities in traditionally handcrafted Shanxi aged vinegar. Through integrated virome and bacteriome analyses, we investigate the structural dynamics of viral communities during acetic acid fermentation, elucidate the tripartite interactions among viruses, bacterial communities, and fermentation environments, and functionally profile viral-encoded genes. This research advances our understanding of viral ecological functions in vinegar brewing processes and provides novel insights into the microbial mechanisms underlying traditional fermented food production.

## 2. Materials and Methods

### 2.1. Sample Collection and Physicochemical Analysis

Vinegar fermentation substrate (vinegar *Pei*) samples of traditionally handcrafted Shanxi aged vinegar during acetic acid fermentation were collected from a traditional vinegar factory (Shanxi Zilin Vinegar Industry Co., Ltd.; Taiyuan, China). The traditional handcrafted Shanxi aged vinegar is fermented in ceramic vats (1 m diameter × 1.2 m height, Supplementary File S1, Figure S1). Vinegar *Pei* samples were collected with slight modifications based on previously described methods [22,31,32]. On Day 1 of acetic acid fermentation, after the first vinegar *Pei*-turnover process, 4 evenly distributed points were selected in the fermentation vat (Supplementary File S1, Figure S1). At each sampling point, the entire vertical column of vinegar *Pei* (from surface to base of the ceramic vat) was collected and homogenized. Subsequently, a 500 g vinegar *Pei* was transferred to a sterile sampling bag, constituting one composite sample. Triplicate vinegar *Pei* samples were collected as biological replicates from three independent ceramic vats on Day 1 of fermentation, systematically labeled as 1d-1, 1d-2, and 1d-3. Identical sampling procedures were performed on Days 5 and 9 of the fermentation process. All samples were immediately transported to the laboratory under cryogenic conditions and stored at −80 °C until nucleic acid extraction and subsequent metagenomic analysis.

The temperature of vinegar *Pei* during acetic acid fermentation was measured at a depth of 30 cm using a calibrated thermometer prior to the first vinegar *Pei*-turnover process in the vinegar factory. The pH of vinegar *Pei* was determined using a pH meter (Delta 320, Mettler Toledo, Greifensee, Switzerland). Specifically, 30 g of fresh vinegar *Pei* was homogenized with triple-volume deionized water (1:3 *w*/*v*), followed by 3 h aqueous extraction at room temperature. The supernatant was then obtained through filter paper filtration prior to pH measurement. The alcohol contents were measured according to the method of Zhang et al. [17]. The acetic acid and lactic acid contents were analyzed as described previously [29].

### 2.2. Viral Purification, Nucleic Acid Extraction, and Sequencing

The viruses in vinegar *Pei* were purified as described previously [22]. Briefly, vinegar *Pei* samples were first pretreated with cold sterile 2% (*w*/*v*) trisodium citrate to stabilize viral particles. At 4 °C, large precipitates were removed by centrifugation at 500× *g* for 10 min, followed by centrifugation at 5000× *g* for 10 min to eliminate cellular debris. The supernatant was diluted with precooled SM buffer (200 mM NaCl, 10 mM MgSO_4_, 50 mM Tris, pH 7.5) and then filtered through 0.22 µm aqueous polyethersulfone membranes to completely remove bacterial impurities. RNase A and DNase I were added to the filtrate to degrade free bacterial DNA and RNA. A sterile 10% (*w*/*v*) PEG8000 (Sigma, St. Louis, MO, USA) aqueous solution was used to precipitate viral particles, which were then concentrated and collected by centrifugation at 12,000× *g*, 4 °C for 1 h. The total viral nucleic acids were extracted using the TIANamp Virus DNA/RNA Kit (Tiangen Biotech, Beijing, China) following the manufacturer’s instructions. To detect the presence of free or contaminating bacterial DNA in the viral concentrate, PCR amplification was performed using the universal bacterial primers 27F (5′-AGAGTTTGATCCTGGCTCAG-3′) and 1492R (5′-GGTTACCTTGTTACGACTT-3′) [33]. The PCR conditions were as follows: initial denaturation at 95 °C for 3 min, followed by 30 cycles (95 °C for 30 s, 53 °C for 30 s, 72 °C for 45 s), with a final extension at 72 °C for 10 min. After PCR, the amplification products were analyzed by agarose gel electrophoresis. The metagenomic shotgun sequencing libraries were constructed and quality-controlled by Shanghai Rongxiang Biotechnology Co., Ltd. (Shanghai, China). High-throughput sequencing was subsequently performed on an Illumina NovaSeq 6000 platform employing a paired-end 150 bp (PE150) sequencing strategy.

### 2.3. Bacterial DNA Extraction and Sequencing

Total genomic DNA was extracted from vinegar *Pei* samples using a plant genomic DNA kit (Tiangen Biotech, Beijing, China) according to the manufacturer’s instructions. The V4 region of the 16S rRNA gene in DNA samples was amplified using specific primers 515F (5′-GTGCCAGCMGCCGCGGTAA-3′) and 806R (5′-GGACTACHVGGGTWTCTAAT-3′) [34]. Following PCR completion, the amplification products were detected by agarose gel electrophoresis. The amplified PCR products sequencing libraries were constructed using the TruSeq Nano DNA LT Library Prep Kit (Illumina, San Diego, CA, USA) according to the manufacturer’s recommendations. Library quality was assessed using the Agilent High Sensitivity DNA Kit (Agilent, Santa Clara, CA, USA) on an Agilent Bioanalyzer (Agilent). Finally, sequencing was performed on an Illumina MiSeq instrument (250 bp, paired-end reads).

### 2.4. Processing of Sequencing Data

For viral metagenomic sequencing data, raw data were trimmed with FASTP (v0.20.0) to remove low-quality reads [35]. Subsequently, MEGAHIT [36] was used for splicing, retaining contigs with a minimum length of 300 bp. Viral contigs were identified using VIBRANT (v2.2.4) and VirSorter2 (v1.2.1), followed by quality control with CheckV. Contigs were further clustered into viral OTUs using CD-HIT (v4.8.1) [37] with thresholds of ≥95% similarity and ≥80% coverage. Taxonomic annotation of the viral OTUs was performed by aligning them against the Virus-NT database using BLASTp (version 2.15.0).

For bacterial amplicon sequencing data, raw sequencing reads were first processed using Cutadapt software (v1.9.3) to remove primer sequences. VSEARCH (v2.13.4_linux_x86_64) software [38] was then employed to cluster high-quality sequences into OTUs at a 97% similarity threshold using the cluster size module. Taxonomic classification of the OTUs was performed against the SILVA database (Release 138) [39] using QIIME2 (2019.4) [40].

### 2.5. Identification of AMGs and ARGs in Viral Metagenomes

The Prodigal software (version 2.6.3) [41] was employed to identify open reading frames (ORFs) of viral contigs. Then the redundancy in protein sequences was reduced using the cluster module of MMseqs2. For functional characterization, the non-redundant protein sequences were aligned against the KEGG and EggNOG databases using the MMseqs2 search module, enabling metabolic pathway reconstruction and Cluster of Orthologous Groups (COG) annotation, respectively. Additionally, carbohydrate-active enzymes (CAZymes) were identified by screening viral ORFs against the CAZyme database using HMMER (v3.1). In addition, antibiotic resistance genes (ARGs) within the virome, were annotated through homology searches against the CARD database using the RGI tool (v.5.1.1) [42].

### 2.6. Statistical Analyses

Alpha diversity indices (Simpson, Chao1, ACE, and Shannon) of bacterial and viral communities were calculated using QIIME to evaluate species richness and evenness. To identify differentially enriched bacterial and viral taxa across fermentation time points, linear discriminant analysis effect size (LEfSe) was performed, with statistical significance thresholds set at an LDA score > 2.0 and *p* < 0.05. Nonmetric multidimensional scaling (NMDS) was employed to assess temporal variations in viral community composition throughout acetic acid fermentation. To investigate potential ecological linkages, genus-level associations between core bacteria and viruses were analyzed using the SparCC algorithm, with stringent correlation thresholds set at *p* < 0.01 and an absolute SparCC value > 0.8. The resulting interaction network was visualized using Cytoscape (v3.8.2). Redundancy analysis (RDA) was performed to examine the associations between fermentation parameters and the community composition of both bacterial and viral populations.

## 3. Results

### 3.1. Virome Sequencing and Fermentation Environmental Dynamics

Nine vinegar *Pei* samples were collected from a traditional Shanxi aged vinegar brewery in Shanxi, China, to investigate the taxonomic and functional diversity as well as the community dynamics of viruses during the acetic acid fermentation process. Viral communities were characterized using metagenomic sequencing. After extracting nucleic acids from the virome and confirming the absence of contaminating bacterial DNA (Supplementary File S1, Figure S2), viral metagenomes from all nine samples were sequenced, and a range of 7.5 to 12.6 Gbp of sequencing data was generated, yielding a range of 45.2 to 74.5 million high-quality reads per virome. De novo assembly yielded an average of 23,513 contigs per sample, with an N50 contig length of 2747 bp. On average, only 2.63% of all contigs showed significant sequence homology to the Virus-NT database. ORF prediction produced an average of 1231 ORFs per vinegar virome (Supplementary File S1, Table S1).

**Figure 1:**
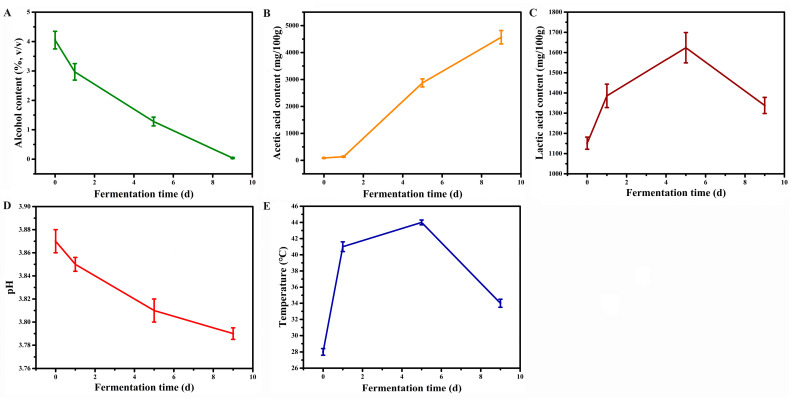
Dynamics of fermentation parameters in the Shanxi aged vinegar acetic acid fermentation process. (**A**) Alcohol. (**B**) Acetic acid. (**C**) Lactic acid. (**D**) pH. (**E**) Temperature. The presented data represent means ± standard deviation from triplicate depth samples collected at identical fermentation time points.

The dynamics of key fermentation parameters during Shanxi aged vinegar fermentation were presented in Figure 1. Acetic acid fermentation is driven primarily by *Acetobacter* species, which oxidize ethanol into acetic acid. The initial alcohol content in the vinegar *Pei* was controlled at around 5% (*v*/*v*). This optimal concentration supports efficient metabolic activity of acetic acid bacteria during fermentation while avoiding alcohol toxicity that could inhibit bacterial viability. During fermentation, a progressive decrease in alcohol content was observed (Figure 1A), accompanied by a corresponding increase in acetic acid content (Figure 1B). Additionally, the fermentation ecosystem also involves lactic acid bacteria (LAB) that produce lactic acid, which modulates the sensory profile by mitigating acetic acid’s sharpness and contributing to a more balanced flavor. Lactic acid concentration exhibited a unimodal trend during fermentation, initially increasing before gradually declining while maintaining concentrations >1150 mg/100 g throughout the process (Figure 1C). The combined accumulation of acetic acid and lactic acid during fermentation progressively decreased the pH of the vinegar *Pei* (Figure 1D). As acetic acid fermentation is an exothermic aerobic process, temperatures were consistently maintained above 40 °C throughout most of the fermentation period. Thermal monitoring revealed that temperature only began to decline during the terminal phase, coinciding with the completion of acetic acid bioconversion (Figure 1E).

### 3.2. Taxonomic Diversities of Viral Community

**Figure 2:**
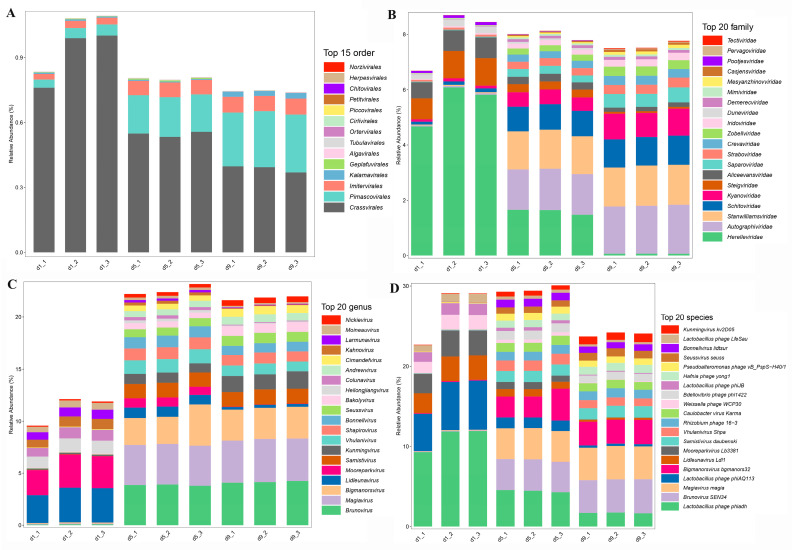
Viral community structure during acetic acid fermentation of Shanxi aged vinegar: (**A**) order level; (**B**) family level; (**C**) genus level; (**D**) species level.

Analysis of the nine Shanxi aged vinegar viromes revealed limited homology to known viral sequences, with only a very small fraction of viral ORFs showing similarity to reference genomes in the Virus-NT database (Figure 2). The vast majority of viral sequences represented novel or uncharacterized viruses, highlighting the extensive unexplored viral diversity in this traditional fermentation ecosystem. Finally, taxonomic analysis of the viral communities identified 16 orders, 53 families, 570 genera, and 1074 species. And all identified viral communities consisted solely of bacteriophages (Figure 2). At the order level, Crassvirales dominated the virome (66.60%). Crassvirales, Pimascovirales, and Imitervirales made up a large fraction of the known viral community (Figure 2A). The family-level composition was primarily represented by Herelleviridae, Autographiviridae, and Stanwilliamsviridae (Figure 2B). At the genus level, Brunovirus, Magiavirus, and Bigmanorsvirus were the predominant genera (Figure 2C). Notably, at the species level, multiple *Lactobacillus* phages were detected, including Lactobacillus phage phiadh, Lactobacillus phage phiAQ113, Lactobacillus phage phiJB, and Weissella phage WCP30 (Figure 2D). This observation aligns with the ecological role of LAB as key microbial taxa in vinegar fermentation. In addition, we identified viruses associated with environmental bacteria, including *Brunovirus* and *Rhizobium* phage, reflecting the open nature of vinegar acetic acid fermentation.

### 3.3. Changes in Viral Communities at the Temporal Scale

**Figure 3:**
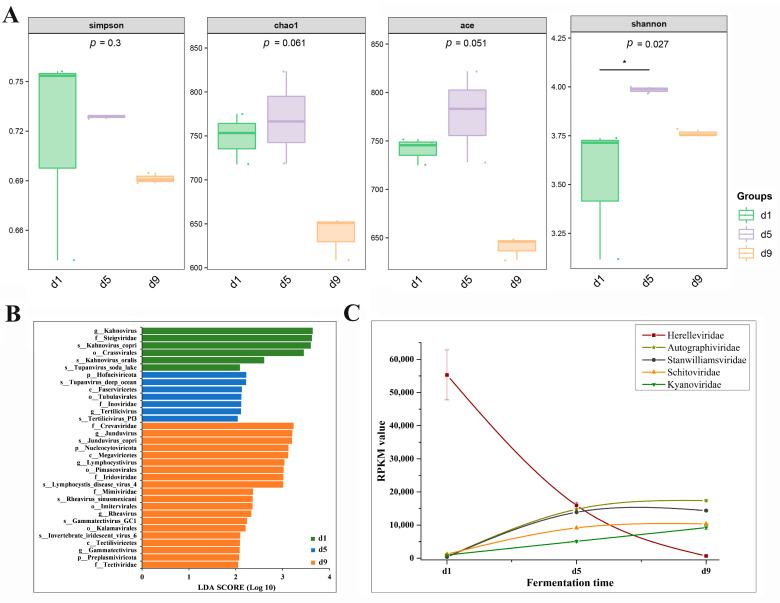
Viral dynamics during the acetic acid fermentation process. Alpha diversity analysis (**A**) and (**B**) LEfSe analysis of viruses along fermentation time (*p* < 0.05 and LDA score > 2.0). (**C**) Abundance of the five predominant viral families along fermentation time, based on RPKM values. * in the figure indicates a statistically significant difference.

To characterize viral community dynamics during acetic acid fermentation, we analyzed the viral community at 3 time points. Non-metric multidimensional scaling (NMDS) was employed to assess temporal variations in viral community structure throughout the acetic acid fermentation process of Shanxi aged vinegar. The analysis revealed clear separation of viral communities across different fermentation time points (Supplementary File S1, Figure S3), demonstrating significant temporal succession. These different clusters in ordination space (stress value = 9.77 × 10^−6^) indicated that viral community composition underwent substantial reorganization as fermentation progressed. Linear regression analysis revealed that the Chao1 and Ace richness and Shannon diversity of the vinegar viral community showed an increasing trend during the early fermentation stage but decreased in the late fermentation phase (Figure 3A). Specifically, Figure 2B revealed significant shifts in viral composition at the family level between the initial fermentation stage (day 1) and the mid-late stages (days 5–9). At day 1, the viral community was predominantly composed of Herelleviridae, Steigviridae, and Aliceevansviridae. This composition underwent substantial transformation by day 5, with Herelleviridae, Autographiviridae, and Stanwilliamsviridae emerging as the dominant families. Further evolution was observed by day 9, when Autographiviridae, Stanwilliamsviridae, and Schitoviridae became the most prevalent viral families.

LEfSe analysis identified 5 orders, 6 families, 6 genera, and 10 species exhibiting significantly different abundances between acetic acid fermentation stages based on RPKM (reads per kilobase per million) values (Figure 3B). Notably, the six genera and six families displayed a one-to-one correspondence. Four bacteriophage families (Mimiviridae, Tectiviridae, Crevaviridae, and Iridoviridae), along with their corresponding four bacteriophage genera, were significantly enriched by day 9 of fermentation, while only one family was enriched on day 1 (Steigviridae) and day 5 (Inoviridae), respectively.

To examine the enrichment dynamics of viruses during acetic acid fermentation, we analyzed the RPKM values of the five predominant viral families (Herelleviridae, Autographiviridae, Stanwilliamsviridae, Schitoviridae, and Kyanoviridae) across different fermentation time points in Shanxi aged vinegar (Figure 3C). From day 1 to day 9 of fermentation, the RPKM value of Herelleviridae decreased significantly, whereas Autographiviridae and Kyanoviridae exhibited a continuous and marked increase. Notably, Stanwilliamsviridae and Schitoviridae showed a dramatic rise in RPKM values during the first 5 days of fermentation, with increases ranging from 647.7% to 3749.9%. Subsequently, from days 5 to 9, the RPKM levels of these two viral families stabilized at a high abundance.

### 3.4. The Relationship Between Viral Communities, Bacterial Communities, and the Fermentation Environment

**Figure 4:**
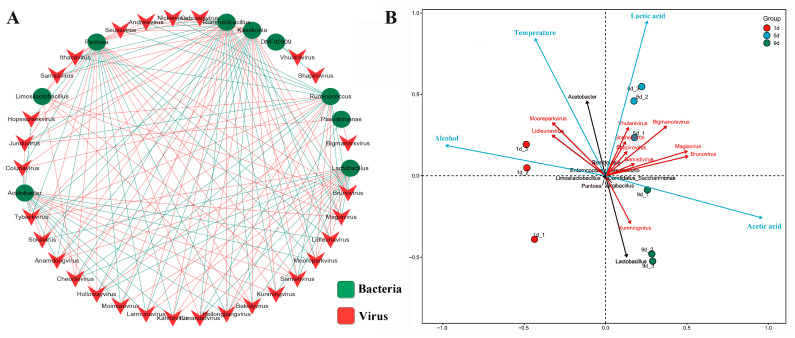
The relationship between viral communities, bacterial communities, and the fermentation environment. (**A**) Association network comprising core bacteria (at the genus level) and dominant viruses (at the genus level) with strict thresholds (*p* < 0.01 and |SparCC value| was >0.8). Edges represent strong positive (red) or negative (green) associations between nodes. (**B**) RDA of viral community dynamics. Vectors show the direction and strength of associations between viruses (red), bacteria (black), and fermentation parameters (blue).

We also analyzed the structure and diversity of bacterial communities in Shanxi aged vinegar during the acetic acid fermentation process. Results showed that *Lactobacillus*, *Acetobacter*, *Romboutsia*, *Enterococcus*, and *Limosilactobacillus* were the top five genera at the genus level, with *Lactobacillus* and *Acetobacter* representing the dominant microbial populations throughout the fermentation process (Supplementary File S1, Figure S4B). The SparCC algorithm was used to further probe the relationship between the core bacteria and dominant viruses at the genus level during acetic acid fermentation of Shanxi aged vinegar with strict thresholds (*p* < 0.01 and |SparCC value| was >0.8). Results showed that 9 bacterial genera and 29 viral genera exhibited strong interconnections, containing 514 links. Notably, viral associations dominated these interactions (67.5% of total links), suggesting potential group effects in viral community responses to environmental stress (Supplementary File S2). Furthermore, virus-bacterium interactions accounted for 27.8% of all links, as visualized in the co-occurrence network (Figure 4A). *Acetobacter* and *Lactobacillus* emerged as the dominant bacterial strains in vinegar fermentation, and the high relative abundance of phages infecting *Acetobacter* and *Lactobacillus* was in line with the dominant existence of their hosts in the vinegar microbiota. Network analysis identified 17 viral genera exhibiting strong associations with the key fermentation strain *Lactobacillus*. Among these, 9 genera (including Samistivirus, Magiavirus, and Brunovirus) displayed positive correlations, while 8 genera (such as Kahnovirus, Larmunavirus, and Tybeckvirus) showed significant negative correlations (*p* < 0.01). Notably, Kahnovirus was the only viral genus significantly enriched on day 1 of fermentation (Figure 3B). In addition, 17 viral genera showed significant associations with *Acetobacter*, comprising 10 positively correlated genera (including Tybeckvirus, Moineauvirus, and Larmunavirus) and 7 negatively correlated genera (such as Hollowayvirus, Magiavirus, and Junduvirus) (*p* < 0.01). Strikingly, Junduvirus displayed stage-specific enrichment, reaching peak abundance exclusively during the terminal fermentation phase (day 9) (Figure 3B). Statistical analysis revealed a strong negative relationship between the dominant genera *Acetobacter* and *Lactobacillus* (SparCC value = −0.97, *p* < 0.01). This bacterial competition was reflected in their associated viral communities. For instance, Magiavirus abundance positively correlated with *Lactobacillus* (SparCC value = 0.87, *p* < 0.01) but negatively with *Acetobacter* (SparCC value = −0.82, *p* < 0.01), while Larmunavirus showed the inverse association pattern (Figure 4A, Supplementary File S2). This demonstrates that the viral community structure is tightly coupled with the dynamics of these key bacterial populations. Notably, several non-dominant bacterial strains maintained extensive viral associations, with *Pantoea* and *Rummeliibacillus* each interacting with 26 viral genera, *Kosakonia* with 23, and *Ruminococcus* with 20. These substantial virus-bacterium interactions may significantly influence the abundance and metabolic activity of their host bacteria.

RDA further unraveled that the relationship among viruses, microbes, and fermentation parameters in Shanxi aged vinegar during the acetic acid fermentation process (Figure 4B). The RDA revealed distinct temporal stratification of viral communities, with clear separation between different fermentation stages. The diversity and abundance of viruses were mainly enriched in the mid-phase of acetic acid fermentation. Most viruses exhibited positive correlations with lactic acid, acetic acid, and temperature, while showing a negative correlation with alcohol content. This suggests that these viruses can persist under specific acidic and thermal conditions but are susceptible to alcohol. In contrast, Lidleunavirus and Mooreparkvirus displayed an inverse relationship, being negatively associated with acetic acid but positively correlated with alcohol levels. Additionally, Kunmingvirus demonstrated negative correlations with both lactic acid concentration and temperature. It should be noted that the changes in the aforementioned environmental factors resulted entirely from the metabolic processes of bacterial communities during fermentation. Therefore, the correlations between viral communities and environmental factors also reflect the underlying interactions between viral and bacterial communities.

### 3.5. Abundant Auxiliary Metabolic Genes of Viruses

Viruses may modulate host metabolic processes through AMGs. To elucidate the functional diversity of viral communities in Shanxi aged vinegar, we performed functional annotation of predicted viral ORFs using the eggNOG database, followed by COG classification (Supplementary File S1, Figure S6). Results revealed that a substantial proportion of viral ORFs remained uncharacterized, indicating the presence of numerous unknown viral genes in the vinegar microbiome. While viral ORFs were annotated across all COG functional categories, the majority were linked to core viral processes, including ‘replication, recombination and repair’ and ‘energy production and conversion’. Intriguingly, beyond these expected functions, the vinegar virome exhibited significant enrichment in ‘carbohydrate transport and metabolism’ (1.0%) and ‘amino acid transport and metabolism’ (1.5%) (Supplementary File S1, Figure S6).

KEGG functional annotations of the virus community were performed by aligning viral ORFs against the KEGG database. Pathway analysis revealed significant enrichment across six level-1 KEGG categories, including ‘cellular processes’, ‘environmental information processing’, ‘genetic information processing’, ‘human diseases’, ‘metabolism’, and ‘organismal systems’ (Supplementary File S1, Figure S5). Notably, approximately 50% of annotated viral genes were associated with metabolic pathways, with particularly high representation in nucleotide metabolism, cofactor/vitamin metabolism, energy metabolism, amino acid metabolism, and carbohydrate metabolism. Amino acid metabolism was able to further generate flavor substances and significantly affect the flavor of fermented foods. Further analysis demonstrated that Shanxi aged vinegar viral AMGs were extensively involved in amino acid metabolic pathways, including ‘alanine, aspartate and glutamate metabolism’, ‘glycine, serine and threonine metabolism’, ‘cysteine and methionine metabolism’, ‘arginine and proline metabolism’, ‘histidine metabolism’, ‘tyrosine metabolism’, ‘phenylalanine, tyrosine and tryptophan biosynthesis’, ‘selenocompound metabolism’, and ‘glutathione metabolism’ (Supplementary File S3). The abundance of viral AMGs associated with amino acid metabolism suggests their potential role in modulating flavor development during Shanxi aged vinegar fermentation. The acetic acid fermentation process is fundamentally driven by the microbial conversion of ethanol to acetic acid, representing a key component of carbohydrate metabolism. KEGG pathway analysis revealed significant viral involvement in this metabolic network through AMGs associated with multiple carbohydrate-processing pathways, including ‘glycolysis/gluconeogenesis’, ‘citrate cycle’, ‘pentose and glucuronate interconversions’, ‘fructose and mannose metabolism’, and ‘galactose metabolism’ pathways (Supplementary File S3).

**Figure 5:**
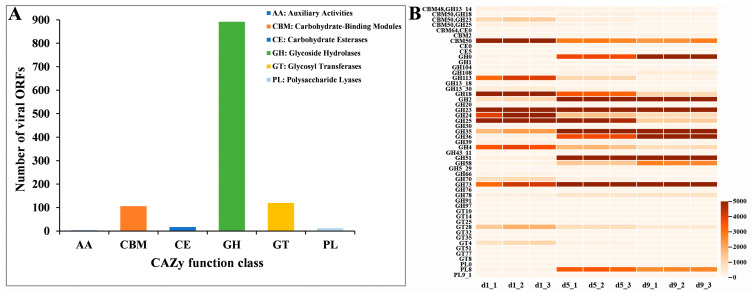
Functional characterization of viral AMGs in vinegar fermentation. (**A**) Relative abundance of CAZyme families in viral genomes. (**B**) Temporal dynamics of CAZyme genes during acetic acid fermentation, shown as a heatmap.

To comprehensively characterize the auxiliary carbohydrate-active genes in vinegar viral communities, we conducted a systematic comparison of virome sequences against the CAZy database. As a result, a total of 1180 ORFs were further identified as CAZymes in vinegar viruses, encompassing key enzymes involved in carbohydrate metabolism, such as alcohol oxidases, cellulase, peptidoglycan lytic transglycosylases, glucosyltransferase, starch phosphorylase, glucan synthase and pectate lyase. Glycoside hydrolases (GHs), glycosyltransferases (GTs) and carbohydrate-binding modules (CBMs) family displayed higher abundances of 76.93%, 10.50% and 9.29% in vinegar viruses, respectively (Figure 5A). Temporal analysis revealed dynamic shifts in CAZyme gene abundance throughout acetic acid fermentation (Figure 5B). Notably, we observed progressive enrichment of several glycoside hydrolase families (GH2, GH35, GH36, GH51, GH58, GH73, GH78, and GH108) and polysaccharide lyase PL8 during fermentation. Particularly significant was the increasing abundance of endoglucanase GH51, which may facilitate the hydrolysis of cellulose present in rice husk—the primary auxiliary material in vinegar fermentation. Conversely, multiple enzyme families displayed decreasing trends, including glycoside hydrolases (GH4, GH18, GH24, GH25, GH39, GH70, and GH113) and glycosyltransferases (GT28 and GT4) (Figure 5B).

### 3.6. Antibiotic Resistance Genes in the Virome

In environmental systems, viruses play a critical role in harboring and transferring antibiotic resistance genes (ARGs) [43]. KEGG pathway analysis revealed significant enrichment of genes associated with ‘Drug Resistance: Antimicrobial’ pathways in the Shanxi aged vinegar virome (Supplementary File S1, Figure S5), suggesting viral communities may facilitate horizontal transfer of ARGs during fermentation. To systematically characterize these ARGs, we aligned viral ORFs against CARD. Our analysis identified 99 distinct ARGs in the vinegar viral metagenome (Supplementary File S4), with 41 ARGs consistently detected across all fermentation stages. These persistent ARGs primarily conferred resistance to fluoroquinolone, aminocoumarin, lincosamide, streptogramin, salicylic acid, diaminopyrimidine, phenicol, peptide, rifamycin (Table 1). Notably, distinct temporal patterns were observed among the most abundant ARGs during fermentation (Supplementary File S4). Specifically, the abundance of Saur_gyrA_FLO, Uure_gyrB_FLO, and vanU_in_vanG_cl showed progressive decline throughout the fermentation process. In contrast, Ngon_parC_FLO and vmlR demonstrated consistent accumulation over time.

## 4. Discussion

### 4.1. Predominant Viral Families in Shanxi Aged Vinegar Fermentation

In the present study, we investigated the taxonomic diversity of viral communities in Shanxi aged vinegar. Among the identified viral families, Herelleviridae exhibited the highest cumulative relative abundance, followed by Autographiviridae, Stanwilliamsviridae, and Schitoviridae. Herelleviridae primarily infect LAB and are frequently detected in fermented dairy products [44]. This is particularly relevant as LAB constitute the dominant fermentative microbiota during the acetic acid fermentation process of Shanxi aged vinegar (Supplementary File S1, Figure S4A). The elevated relative abundance of LAB-infecting viruses was in line with the predominance of their bacterial hosts in the vinegar microbiome. Furthermore, Herelleviridae were consistently detected throughout Baijiu fermentation, including both the initial *Daqu* production stage and subsequent main fermentation processes [18,45]. In Shanxi aged vinegar production, solid-state alcohol fermentation precedes acetic acid fermentation, with all alcohol fermentation products serving as substrates for the subsequent acetic fermentation [24]. Notably, Herelleviridae abundance peaked on day 1 of acetic fermentation and gradually declined thereafter (Figure 2B), suggesting these viruses were primarily derived from the preceding alcohol fermentation stage.

Additionally, we previously characterized the viral community structure in Zhenjiang aromatic vinegar, another traditional grain vinegar in China [22]. Comparative analysis revealed significant differences between the viral communities during acetic acid fermentation in these two solid-state fermentation vinegars. First, regarding viral diversity, Shanxi aged vinegar exhibited greater diversity, with 53 viral families and 570 genera identified, compared to 40 families and 258 genera in Zhenjiang aromatic vinegar. Second, the viral community composition differed markedly between the two vinegar types. Shanxi aged vinegar was dominated by the families Herelleviridae, Autographiviridae, and Stanwilliamsviridae, whereas Zhenjiang aromatic vinegar was primarily characterized by Myoviridae, Siphoviridae, and Caudovirales [22]. Notably, while several families (including Mimiviridae, Ackermannviridae, Phycodnaviridae, and Inoviridae) were shared between both vinegar types, key dominant families in Shanxi aged vinegar (Herelleviridae, Autographiviridae, Stanwilliamsviridae, and Schitoviridae) were completely absent in Zhenjiang vinegar samples. These distinct viral profiles likely reflect fundamental differences in both fermentation substrates and production processes between these two traditional solid-state fermentation vinegars. Shanxi aged vinegar utilizes solid sorghum alcohol fermentation mash (containing partially fermented sorghum and bran) along with wheat chaff as raw materials for acetic acid fermentation. It proceeds directly without additional inoculation, employing a dynamic solid-state fermentation process that requires frequent and thorough mixing of all mash materials throughout fermentation [24]. In contrast, Zhenjiang aromatic vinegar employs liquid glutinous rice wine mash, bran, and rice husks as its base. It requires the addition of a starter culture for fermentation and adopts a layered fermentation method during the process [25]. Comparative virome analysis revealed striking differences between the viral communities in solid-state fermented Shanxi aged vinegar and liquid-fermented rice vinegar. Previous studies identified Peduoviridae, Straboviridae, Casjensviridae, and Drexlerviridae as the dominant viral families in liquid-fermented rice vinegar [23]. Although these families were also detected in Shanxi aged vinegar, they exhibited significantly lower relative abundances, which may be primarily due to the significant differences in the brewing processes between solid-state fermented vinegar and liquid-state fermented vinegar. The above comparison reveals that the viral community structures in vinegar produced by different fermentation methods exhibit significant niche differentiation. This enables the formation of geographically distinct virome fingerprints for vinegar, which, compared to their host bacterial communities, can more accurately be used for vinegar identification, quality control, and traceability [27,28].

### 4.2. Viral Communities Are Closely Associated with Bacterial Communities and the Fermentation Environment

During the acetic acid fermentation of Shanxi aged vinegar, both bacterial and viral community structures underwent significant dynamic changes. As fermentation progressed, bacterial diversity showed a gradual decreasing trend (Supplementary File S1, Figure S4C). The dominant viral families shifted dynamically during fermentation. At the initial fermentation stage, the viral community was predominantly composed of Herelleviridae, Steigviridae, and Aliceevansviridae. This composition shifted to Herelleviridae, Autographiviridae, and Stanwilliamsviridae at the mid-fermentation stage. At the final fermentation stage, viral families were dominated by Autographiviridae, Stanwilliamsviridae, and Schitoviridae (Figure 2B). Additionally, close interconnections were observed between viral and bacterial communities. Association network analysis revealed robust interactions between 9 bacterial genera and 29 viral genera, forming 143 virus-bacteria links. Network analysis identified 17 viral genera exhibiting strong associations with the key fermentation strain *Lactobacillus*. In addition, 17 viral genera showed significant associations with *Acetobacter*. Interestingly, even non-dominant bacterial strains exhibited extensive viral connectivity. Specifically, *Pantoea* demonstrated strong associations with 26 viral genera, while *Kosakonia* showed close links to 23 viral genera (Figure 4A). Both *Pantoea* and *Kosakonia* exhibited relatively low abundance levels, which showed a continuous decline throughout the fermentation process (Supplementary File S1, Figure S4D). This observed reduction may be attributed to viral lysis, with the resultant lysates potentially providing essential nutrients for the primary fermentation strains *Acetobacter* and *Lactobacillus*. This phenomenon was also discovered in the study of kimchi fermentation, where phage-mediated lysis of *Weissella cibaria* was shown to promote the growth of another fermentation strain, *Leuconostoc citreum* [46]. The interconnections between bacterial and viral communities were associated with the numerous defense systems present in their respective genomes, including toxin-antitoxin systems and CRISPR-Cas systems [47]. This has been demonstrated in liquid-fermented rice vinegar, where studies revealed that toxin-antitoxin systems (including 19 types of toxins and 15 types of antitoxins) and four incomplete CRISPR-Cas systems were identified in the viral community genomes of rice vinegar [23]. Moreover, the infection of host bacteria by phages is related to the host’s physiological state. For example, when *Vibrio cholerae* transitions from a viable but non-culturable (VBNC) state to a culturable state, the expression of phage shock proteins (*pspA*, *pspB*, and *pspC*) increases [48]. Additionally, bacteriophage K exhibits different infectivity capabilities against *Staphylococci* in the VBNC state compared to those in the culturable state [49].

In the present study, the viral community in Shanxi aged vinegar also showed significant associations with fermentation environmental factors. Most viruses were positively correlated with lactic acid and acetic acid concentrations, as well as temperature, but negatively correlated with alcohol content. In contrast, *Lidleunavirus* and *Mooreparkvirus* exhibited negative correlations with acetic acid levels and positive correlations with alcohol content (Figure 4B). A similar phenomenon has been observed in cider fermentation, where the infectivity and stability of viruses targeting lactic acid bacteria were temperature-dependent. Compared to fermentation at 15 °C, the presence of exogenous phages at a relatively higher temperature (25 °C) contributed to the stability of the fermenting microbial community, thereby ensuring cider quality [50]. Another aspect of environmental influence on viral communities may lie in their ability to induce lysogenic conversion, thereby affecting bacterial populations. For example, nitrogen availability in soil can modulate viral lysogeny, with short-term urea eutrophication favoring a lysogenic strategy in viruses [51]. Similarly, a phage in *Pseudomonas* sp. regulates the lysis–lysogeny switch in response to chitin availability [5]. Temperate phages can drive mutualistic phage-host interactions through lysogenic conversion [52]. In this study, the dynamic changes in environmental factors during fermentation, such as acidity, alcohol content, and temperature, are likely to influence the virus lysis–lysogeny switch, consequently shaping the microbial community structure in vinegar fermentation. These findings regarding the relationship between bacterial and viral communities significantly enhance our understanding of the viral ecology in Shanxi aged vinegar fermentation and provide fundamental data for future research.

### 4.3. Abundant Functional Genes in the Viral Genome

Research findings demonstrated that viruses present in fermented foods played a stabilizing role in fermentative bacterial communities [50]. The functional genes acquired by viruses through horizontal gene transfer (HGT) from host genomes significantly contribute to virus–host coevolution, thereby enhancing the adaptive capacity of both viral and bacterial populations to environmental fluctuations [53,54]. One significant outcome of HGT is the acquisition of AMGs by the virome. In this study, we identified numerous AMGs associated with amino acid metabolism, including genes for ‘alanine, aspartate and glutamate metabolism’, ‘glycine, serine and threonine metabolism’, and ‘cysteine and methionine metabolism’ (Supplementary File S3). Additionally, we detected abundant auxiliary carbohydrate metabolic genes in the vinegar viral community, with GHs constituting the predominant family (76.93%) (Figure 5A). In the viral community of Zhenjiang aromatic vinegar (also solid-state fermented), a substantial number of auxiliary carbohydrate metabolic genes were also identified. However, unlike our current findings, GTs, GHs, and carbohydrate esterases (CEs) were all prominently represented in the Zhenjiang vinegar virome [22]. By contrast, in liquid-fermented rice vinegar, GTs and GHs dominated the profile [23]. These differences likely stem primarily from variations in production techniques and environmental conditions, which consequently shape distinct viral community structures. In addition to vinegar, substantial carbohydrate metabolic genes have also been identified in the viromes of other fermented foods such as Baijiu and soy sauce [19,45], which likely was due to the high carbohydrate content characteristic of these fermentation substrates. Viruses can regulate host metabolism through encoded AMGs [55]. Phages in aquatic environments could promote intracellular sulfur and thiosulfate oxidation/disproportionation metabolic processes in host bacteria by encoding sulfur metabolism-related AMGs [2]. The prophage-encoded chitinase facilitated chitin degradation and promoted the growth of host *Pseudomonas* sp. in chitin-containing environments [5]. Similarly, the abundant viral AMGs associated with carbohydrate and amino acid metabolism in vinegar fermentation might likely participate in both the saccharification of complex polysaccharides and the development of characteristic flavors in Shanxi aged vinegar.

In addition to AMGs, another consequence of HGT is the acquisition of ARGs by viral genomes. Viruses have long been recognized as reservoirs of antibiotic resistance genes in environmental settings [43,56]. Our viral metagenomic analysis revealed an extensive ARG repertoire in vinegar fermentation, with 99 identified ARGs conferring resistance to nine antibiotic classes: fluoroquinolones, aminocoumarins, lincosamides, streptogramins, salicylic acid, diaminopyrimidines, phenicols, peptide antibiotics, and rifamycins (Table 1). On one hand, this is related to the production materials, as both bran and rice husk, the raw materials for acetic acid fermentation, have been reported to contain antibiotics [57,58]. On the other hand, it may be due to the open fermentation process of acetic acid, where bacteria carrying ARGs from the environment could contaminate the fermentation. Similarly, ARGs have also been detected in the fermentation of Zhenjiang aromatic vinegar, though at significantly lower levels (*n* = 29) [22]. This discrepancy may be attributed to variations in production processes and environmental conditions or to the amount of sequencing. The antibiotic resistance genes carried by viruses enhance the host’s environmental adaptability, enabling resistance to residual antibiotics in the fermentation raw materials, such as bran and rice husk [57,58]. In summary, the viruses in Shanxi aged vinegar fermentation broth influence host metabolism through functional genes like AMGs and ARGs, thereby promoting the adaptability of both viruses and hosts to the dynamic fermentation environment.

## 5. Conclusions

In this study, we characterized the composition and functional potential of viral communities during the acetic acid fermentation of Shanxi aged vinegar. Metagenomic analysis revealed that Herelleviridae, Autographiviridae, and Stanwilliamsviridae were the three most dominant viral families, and all identified viral families were bacteriophage families, with viral community structure and diversity undergoing significant shifts as fermentation progressed. The viral communities in vinegar exhibited strong correlations with bacterial communities and demonstrated significant interactions with environmental factors. Additionally, viral communities harbored diverse AMGs related to carbohydrate and amino acid metabolism, which may participate in host metabolic processes. However, this work only provides initial insights into virus-bacteria interactions during vinegar fermentation; several questions remain unresolved. Future studies should focus on the following: (1) the interactions between viruses and fungal communities; (2) functional validation of abundant AMGs in viruses; (3) conditions triggering the viral lytic–lysogenic switch; and (4) the impact of these factors on the flavor quality of vinegar. These investigations are crucial for comprehensively elucidating the role of viral communities in the fermentation process of Shanxi aged vinegar.

## Figures and Tables

**Table 1:** List of ARGs retrieved from vinegar virome contigs (top 20 by abundance).

Protein ID	ARO Accession	Drug Class	ARG Name	*e* Value
gene_679	ARO:3003296	Fluoroquinolone antibiotic; nybomycin-like antibiotic	Saur_gyrA_FLO	1.14 × 10^−62^
gene_1866	ARO:3004835	Carbapenem; Cephalosporin; Cephamycin; Monobactam; Penam	Ngon_pilQ_BLA	1.36 × 10^−107^
gene_1871	ARO:3004833	Carbapenem; Cephalosporin; Cephamycin; Monobactam; Penam	Ngon_PBP1_BLA	2.41 × 10^−194^
gene_2060	ARO:3003931	Fluoroquinolone antibiotic; Nybomycin-like antibiotic	Cgin_gyrA_FLO	4.80 × 10^−31^
gene_2064	ARO:3003305	Aminocoumarin antibiotic; Fluoroquinolone antibiotic	Uure_gyrB_FLO	1.12 × 10^−22^
gene_2223	ARO:3003929	Fluoroquinolone antibiotic	Ngon_parC_FLO	4.30 × 10^−22^
gene_2751	ARO:3004476	Lincosamide antibiotic; Streptogramin B antibiotic; Streptogramin antibiotic	vmlR	6.16 × 10^−9^
gene_4728	ARO:3004480	Peptide antibiotic; Rifamycin antibiotic	Bado_rpoB_RIF	1.86 × 10^−10^
gene_6593	ARO:3004153	Salicylic acid antibiotic	Mtub_thyA_PAS	5.78 × 10^−59^
gene_13680	ARO:3003296	Fluoroquinolone antibiotic; Nybomycin-like antibiotic	Saur_gyrA_FLO	1.06 × 10^−61^
gene_22061	ARO:3007051	Fluoroquinolone antibiotic; Peptide antibiotic; Rifamycin antibiotic	Hpyl_rpoB_RIF	3.89 × 10^−9^
gene_27239	ARO:3003305	Aminocoumarin antibiotic; Fluoroquinolone antibiotic	Uure_gyrB_FLO	1.39 × 10^−60^
gene_31692	ARO:3004476	Lincosamide antibiotic; Streptogramin B antibiotic; Streptogramin antibiotic	vmlR	1.97 × 10^−9^
gene_34004	ARO:3003463	Disinfecting agents and antiseptics; Isoniazid-like antibiotic	Mtub_kasA_INH	1.81 × 10^−6^
gene_34452	ARO:3004153	Salicylic acid antibiotic	Mtub_thyA_PAS	4.51 × 10^−71^
gene_43223	ARO:3000813	Diaminopyrimidine antibiotic; Fluoroquinolone antibiotic; Phenicol antibiotic	MexS	7.53 × 10^−8^
gene_45120	ARO:3003307	Aminocoumarin antibiotic; Fluoroquinolone antibiotic	Sser_gyrB_FLO	1.24 × 10^−39^
gene_48161	ARO:3007051	Fluoroquinolone antibiotic; Peptide antibiotic; Rifamycin antibiotic	Hpyl_rpoB_RIF	2.32 × 10^−10^
gene_48207	ARO:3004253	Glycopeptide antibiotic	vanU_in_vanG_cl	3.48 × 10^−15^
gene_50049	ARO:3003305	Aminocoumarin antibiotic; Fluoroquinolone antibiotic	Uure_gyrB_FLO	1.42 × 10^−66^

## Data Availability

The raw data of bacterial and viral metagenomes are available at NCBI Sequence Read Archive (NCBI SRA) under BioProject accession no. PRJNA1295169.

## Supplementary Materials

The following supporting information can be downloaded at: https://www.mdpi.com/article/10.3390/foods14173095/s1. Supplementary File S1: Table S1 and Figure S1–S6; Table S1. Statistical analysis and processing of viral metagenomic sequencing data from Shanxi aged vinegar; Figure S1. Sampling of vinegar Pei from the fermentation vat of Shanxi aged vinegar. Four evenly distributed points were selected in the fermentation vat. At each sampling point, the entire vertical column of vinegar Pei (from surface to base of the ceramic vat) was collected and homogenized. Figure S2. Agarose gel electrophoresis of PCR amplification products of virome nucleic acid extracts using the universal bacterial primers 27F/1492R. (a) Lane 1: DNA marker; Lane 2: bacterial positive control; Lanes 3–5: d1 samples; Lanes 6–8: d5 samples. (b) Lane 1: DNA marker; Lane 2: bacterial positive control; Lanes 3–5: d9 samples. Figure S3. NMDS assess temporal variations in viral community structure throughout the acetic acid fermentation process of Shanxi aged vinegar. Figure S4. Bacterial community structure and dynamics during acetic acid fermentation of Shanxi aged vinegar. A: Family level; B: Genus level; C: Alpha diversity analysis of bacteria community along fermentation time; D: Heatmap of bacterial community dynamics. Figure S5. KEGG pathway classification of viral metabolic genes. Figure S6. COG functional categories of viral genes. Supplementary File S2: SparCC correlations; Supplementary File S3: KEGG pathway summary at L3 level; Supplementary File S4: Antibiotic resistance genes in the vinegar virome.
