# Supplementary material for: Insights into the Composition and Function of Virus Communities During Acetic Acid Fermentation of Shanxi Aged Vinegar
Source: Foods. 2025 Sep 3;14(17):3095. doi: 10.3390/foods14173095 (PMC12428672; doi:10.3390/foods14173095)
Supplement: Supplementary file 1 [file foods-14-03095-s001.zip › Supplementary File S1.pdf]

Insights into the composition and function of virus communities during acetic acid fermentation of Shanxi aged vinegar

Zhen Yu[1,2], Xujiao Zhang[1], Tingting Ma[2], Huizi Zhao[1], Yufeng Yan[1], Yongjian Yu[2*]

[1] Shanxi Provincial Key Laboratory for Vinegar Fermentation Science and Engineering, Shanxi Zilin Vinegar Industry Co., Ltd., Taiyuan 030400, China

[2] School of Grain Science and Technology, Jiangsu University of Science and Technology, Zhenjiang 212003, China

Table S1. Statistical aanalysis and processing of viral metagenomic sequencing data from Shanxi aged vinegar

Samples	Total_bases	Total reads	Number of clean reads	Number of contigs	N50 of contigs	Proportion of contigs showing homology to the Virus-NT database (%)	Predicted ORF number
1d-1	10265719800	68438132	63697982	19551	4014	3.36	1169
1d-2	8940729900	59604866	54196586	18615	3078	3.48	1153
1d-3	7452984900	49686566	45225986	16280	2495	3.58	1160
5d-1	9740915100	64939434	58637896	26580	3177	2.48	1355
5d-2	10173513600	67823424	61044714	26826	3069	2.10	1308
5d-3	7617694200	50784628	47573992	24993	2437	2.32	1291
9d-1	9668453700	64456358	58070330	26809	1896	2.04	1162
9d-2	9153309000	61022060	53298256	26194	1811	2.06	1169
9d-3	12577092900	83847286	74535708	25772	2746	2.22	1320

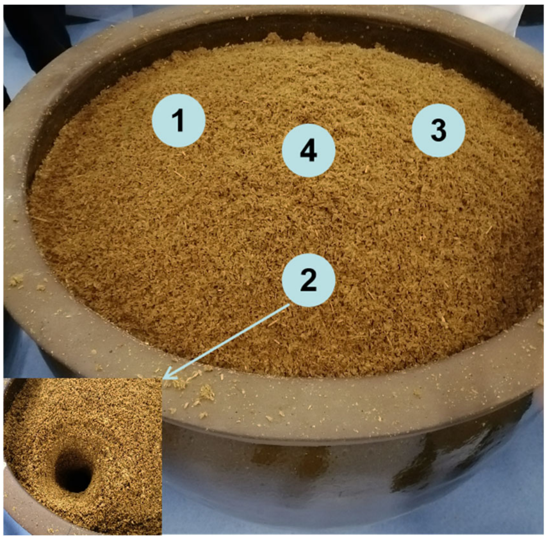

Figure S1. Sampling of vinegar *Pei* from the fermentation vat of Shanxi aged vinegar. Four evenly distributed points were selected in the fermentation vat. At each sampling point, the entire vertical column of vinegar *Pei* (from surface to base of the ceramic vat) was collected and homogenized.

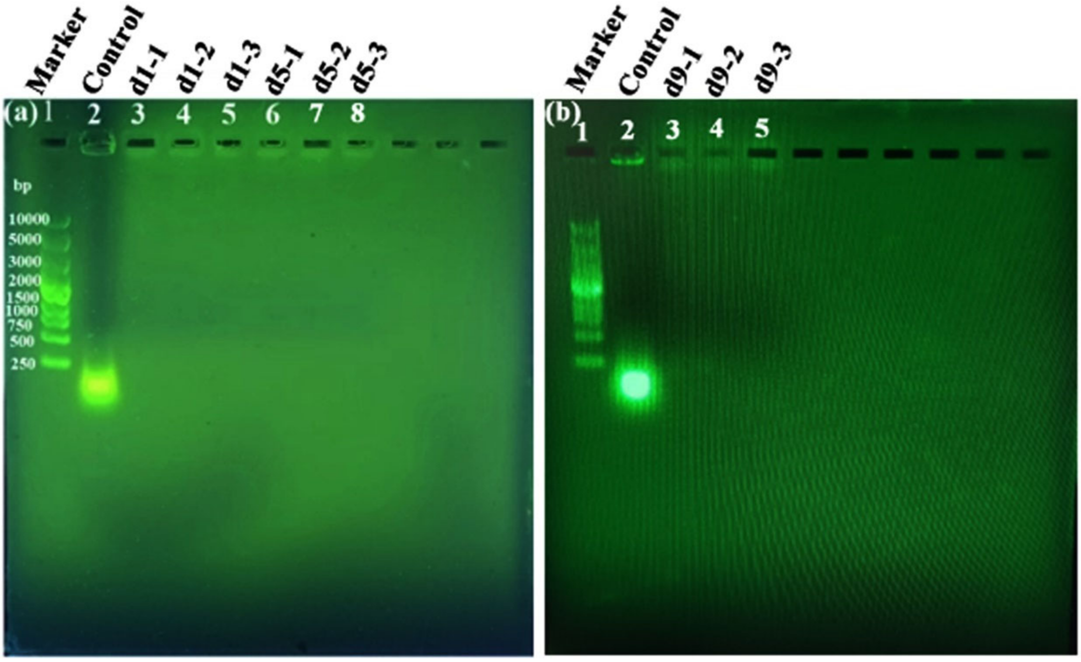

Figure S2. Agarose gel electrophoresis of PCR amplification products of virome nucleic acid extracts using the universal bacterial primers 27F/1492R. (a) Lane 1: DNA marker; Lane 2: bacterial positive control; Lanes 3–5: d1 samples; Lanes 6–8: d5 samples. (b) Lane 1: DNA marker; Lane 2: bacterial positive control; Lanes 3–5: d9 samples.

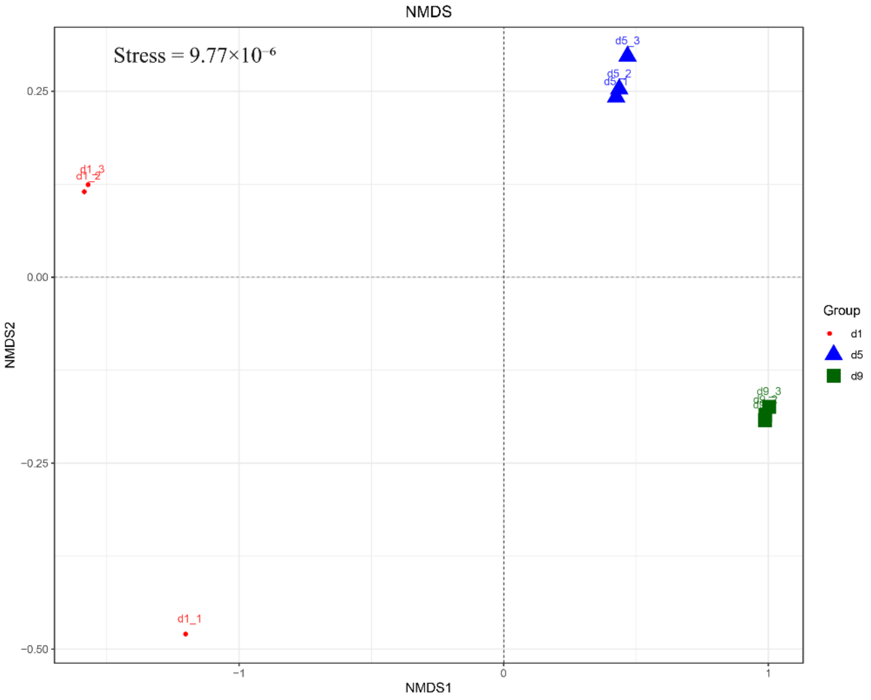

Figure S3. NMDS assess temporal variations in viral community structure throughout the acetic acid fermentation process of Shanxi aged vinegar.

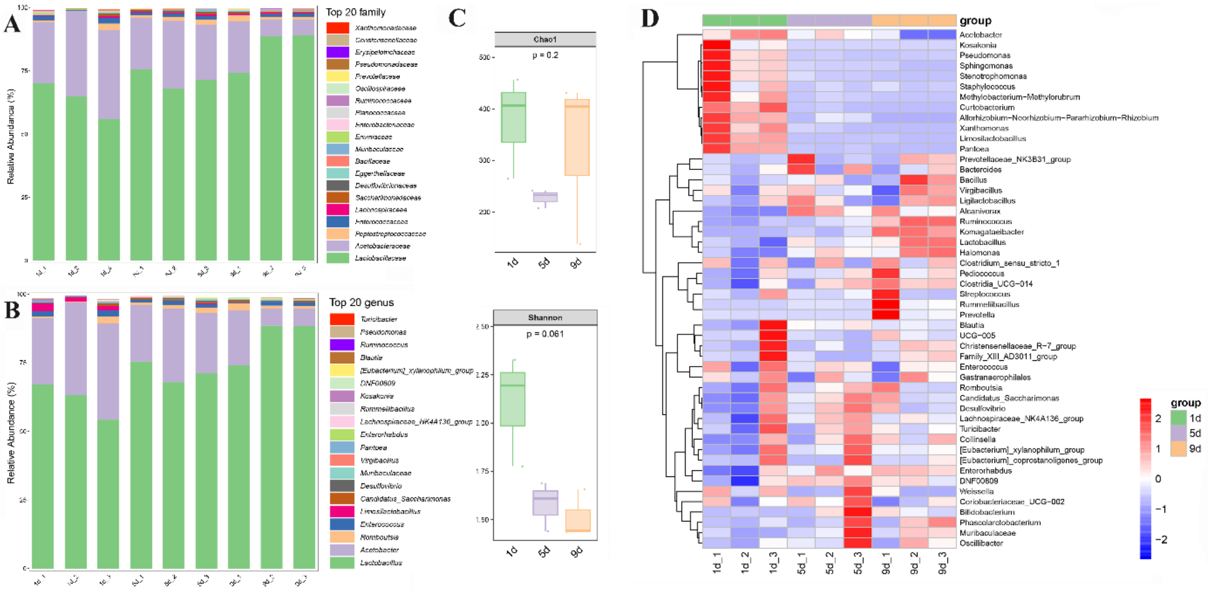

Figure S4. Bacterial community structure and dynamics during acetic acid fermentation of Shanxi aged vinegar. A: Family level; B: Genus level; C: Alpha diversity analysis of bacteria community along fermentation time; D: Heatmap of bacterial community dynamics.

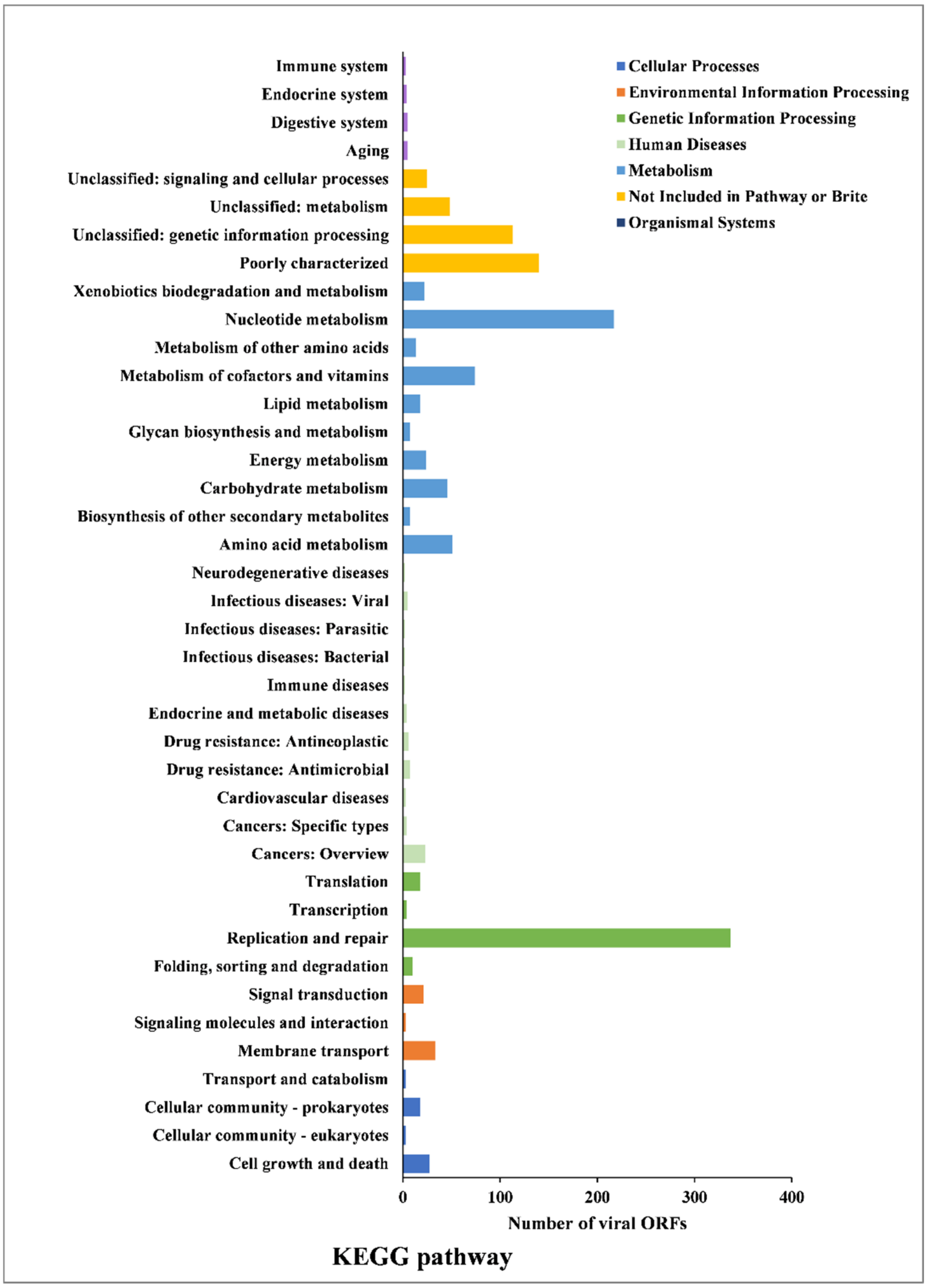

Figure S5. KEGG pathway classification of viral metabolic genes.

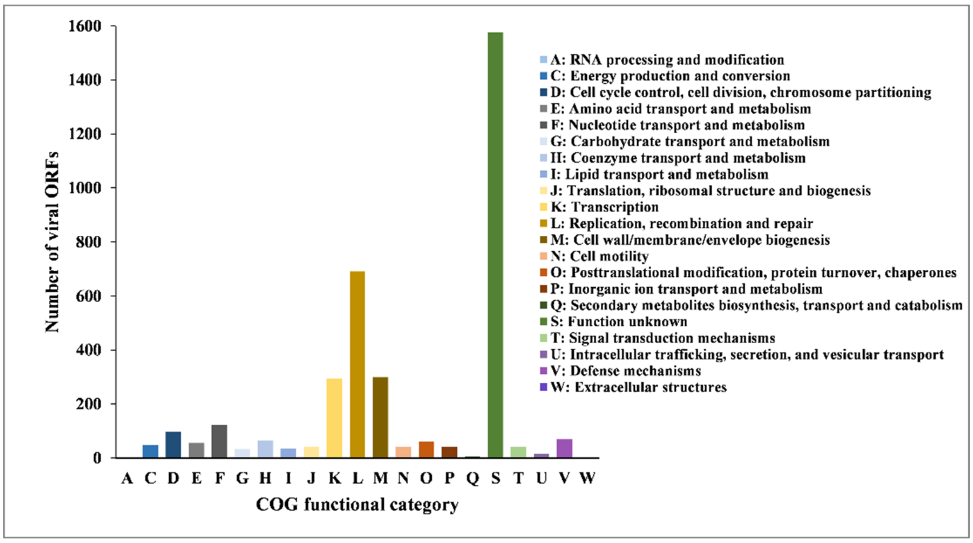

Figure S6. COG functional categories of viral genes
